# Combined effects of age and gender on gait symmetry and regularity assessed by autocorrelation of trunk acceleration

**DOI:** 10.1186/1743-0003-11-109

**Published:** 2014-07-04

**Authors:** Hiromitsu Kobayashi, Wataru Kakihana, Tasuku Kimura

**Affiliations:** 1 Department of Nursing, Ishikawa Prefectural Nursing University, 1-1 Gakuendai, Kahoku, Ishikawa 929-1212, Japan; 2 University Museum, University of Tokyo, 7-3-1, Hongo, Bunkyo, Tokyo 113-0033, Japan

**Keywords:** Accelerometry, Symmetry, Regularity, Gait, Autocorrelation

## Abstract

**Background:**

The gait of a healthy person is believed to be more regular and symmetrical than those of an individual with a disease. Thus, symmetry and regularity are important indicators of human gait.

**Methods:**

The effects of age and gender on gait symmetry and regularity were investigated in 87 Japanese participants by measuring trunk accelerometry during a 7-m walk. The younger group included 26 female and 21 male students, and the elderly group included 24 females and 16 males. Average age for each group was 20 and 70 years, respectively. Gait symmetry and regularity were evaluated on the basis of autocorrelation functions of trunk accelerations of vertical and anteroposterior axes.

**Results:**

The relationship between age and gait symmetry and regularity was statistically significant for both vertical and anteroposterior axes. Elderly participants showed lower symmetry and regularity in their gait than young participants. A significant gender effect was observed for the symmetry index of both axes but not for the regularity index. Male participants showed lower gait symmetry than females. An interaction effect between age and gender was significant in the symmetry index of anteroposterior acceleration. Gender effect was appeared more clearly in elderly than young participants.

**Conclusion:**

Elderly participants showed a more asymmetrical and irregular gait than young participants. In addition to age, a significant gender effect was observed on gait symmetry. However, the effect size of gender was smaller than that of age, and it was not significant for gait regularity. The gait indices obtained by autocorrelation of trunk acceleration can be considered useful to evaluate aging effect on gait.

## Background

Gait symmetry and regularity may vary with the individual health condition. Although a healthy person’s gait is more regular and symmetrical than the gait of an individual with a disease. Marked irregularity in gait has been reported in patients with Parkinson’s disease [1], and asymmetrical gait was observed in patients with limb-length discrepancy [2], amputees [3], patients with hip osteoarthritis [4], and in patients with chronic stroke [5]. While these studies examined symmetry and/or regularity in patients with orthopedic, neurological, or cerebrovascular diseases, there is little information on the gait characteristics of the normal population.

Age is known to have an effect on gait, with young people walking faster, with longer steps and a higher step frequency compared with elderly people [6]. These changes in gait could be attributed to decline of physical and neurological functions with aging. Similarly, gait symmetry and regularity also are considered to depend on physical and neurological functions [7]; therefore, decreased symmetry and regularity can be expected in the elderly. Furthermore, previous studies have demonstrated different effect of age on some gait characteristics between males and females [8,9]; thus an interaction of age and gender on gait symmetry and regularity can be assumed.

Gait symmetry and regularity can be calculated from various gait features including spatial [10,11], temporal [10-12], kinetic [10,13], or kinematic [14,15] features. In the late 1990s Moe-Nilssen suggested a simple new approach to measure gait symmetry and regularity on the basis of autocorrelation of trunk acceleration [16,17]. In recent years, the utility of this method in gait analysis has been expanded. The present study evaluated gait symmetry and regularity in the normal Japanese population and examined the effect of age and gender on the gait features by autocorrelation of trunk acceleration.

## Methods

### Participants

Data was gathered from 40 community-dwelling elderly people (24 female, 16 male) and 47 younger university students (26 female, 21 male), with average ages of 70 and 20 years for each group, respectively. The elderly participants were active enough to visit our experimental site in Kahoku City, Ishikawa, Japan. None of the elderly participants were inpatients of hospitals or residents of long-term health care centers. Participant’s age, height, and body mass data are shown in Table 1. The study was approved by the Institutional Ethical Committee of the Ishikawa Prefectural Nursing University, Japan. All the participants provided written informed consent after the aim and procedure of the experiment were explained to them.

**Table 1:** Participant’s demographics

**Age**	**Gender**	**n**	**Age (year)**		**Height (cm)**		**Body mass (kg)**		**BMI (kg/m**^**2**^**)**	
			**Mean**	**SEM**	**Mean**	**SEM**	**Mean**	**SEM**	**Mean**	**SEM**
Elderly	Female	24	69.8	0.5	150.9	0.9	54.9	1.3	24.1	0.4
	Male	16	71.5	0.9	165.4	2.1	65.8	2.6	24.0	0.7
Young	Female	26	20.9	0.3	158.7	1.3	52.3	1.4	20.8	0.5
	Male	21	20.3	0.4	173.2	1.5	66.8	2.5	22.3	0.8

### Measurements and procedures

Acceleration while walking was measured using a triaxial accelerometer (AC-301; GMS Inc., Tokyo, Japan), with a recording interval of 0.02 s (50 Hz). Acceleration was measured within a range of ±2 g. The accelerometer was tightly attached to the waist at the back (at approximately L3–L4). The participants were instructed to walk barefoot at their preferred speed for 7 m and return over a walkway set up in a gymnasium with wooden flooring. All participants walked the 7-m walkway in approximately 5–8 s. The entire procedure was consecutively repeated twice. Thus, the participants walked the 7-m course four times.

### Autocorrelation function

In this study, gait indices were obtained from the autocorrelation function of a series of acceleration measurements. An autocorrelation function defines the correlation between the value at a time t = i and the value at t = i + j, with j representing a time lag. If the autocorrelation is obtained from a finite length signal, it attenuates as the lag increases. Autocorrelation values peak once for each step cycle, and therefore peak values depend on cadence. The autocorrelation function thus follows the equation:

(1) $$R\left(j\right)=\frac{1}{N-j}\sum_{i=1}^{N-j}v\left(i\right)v\left(i+j\right)$$

where R(j) is the autocorrelation function with a time-lag of j, v(i) is the i-th data and N represents the sample size. In the case of j = 0, autocorrelation is calculated using the following equation:

(2) $$R\left(0\right)=\frac{1}{N}\sum_{i=1}^{N}v{\left(i\right)}^{2}$$

Moe-Nilssen and Helbostada [18] refer to this equation as an unbiased autocorrelation. In this study, the autocorrelation was normalized by dividing the time-lagged value by the R(0), according to the following the equation:

(3) $$R'\left(j\right)=\frac{R\left(j\right)}{R\left(0\right)}$$

**Figure 1:**
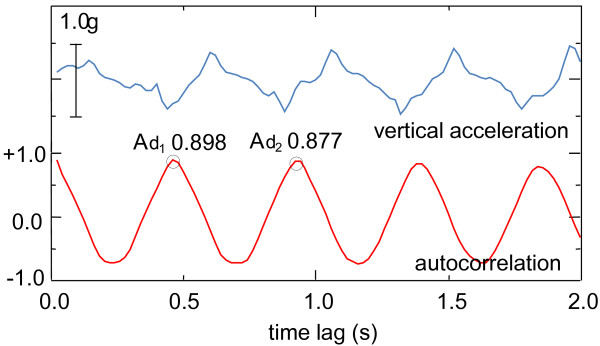
**Signals for vertical acceleration and their autocorrelation.** The vertical accelerations signal (upper line) shows a periodic fluctuation in accordance with the step cycle. The autocorrelation function (lower line) also indicates a periodic fluctuation with the same period. The values of each peak (Ad1 and Ad2) provided indices of symmetry and regularity, respectively.

This normalized autocorrelation varies from −1 to +1, becoming 1 when there are no differences between the time-lagged correlation and the static correlation. As the nonperiodic component was eliminated from the raw signal data, only a smooth fluctuation in the autocorrelation was detected. The autocorrelation indicated a cyclical change, with the period of the cycle coinciding with the step interval. The first peak of autocorrelation (Ad_1_, Figure 1) indicates a correlation between steps and is therefore considered the symmetry index. In addition, because the second peak (Ad_2_, Figure 1) represents a correlation between a stride and the next one, the second peak of the autocorrelation can be considered as the regularity index. Note that in previous studies [19-21], Ad_1_ and Ad_2_ were described as step regularity and stride regularity respectively. Moreover, regardless the axis (vertical, anteroposterior, or mediolateral) used for calculating the autocorrelation function, the symmetry index (Ad_1_ of the autocorrelation) means a correlation between the left and right steps.

The autocorrelation function for anteroposterior acceleration shows an almost similar pattern to that of vertical acceleration, suggesting that symmetry and regularity indices can in principle be obtained either way. However, an autocorrelation of mediolateral acceleration indicates a different pattern. Ad_1_ indicates negative peak, whereas Ad_2_ indicates a positive peak similar to the other axes. In this study, mediolateral acceleration was not used for analysis because it’s autocorrelation indicated unstable form in some cases. Thus, symmetry and regularity indices for the vertical (SI-Vt and RI-Vt) and for anteroposterior (SI-Ap and RI-Ap) accelerations were analyzed in this study.

The autocorrelation function was computed using data from the 4-s mark (after an average of approximately 6 steps) in the middle of each 7-m walk. The results of the four measurements were averaged for further analysis. The expected reproducibility of the average of the four measurements (ICC [1,4]) were 0.860, 0.833, 0.818, and 0.692 for the SI-Vt, RI-Vt, SI-Ap, and RI-Ap, respectively. These ICC values were almost identical to those found in the previous study [22], thus the measurement of the present study considered to be reliable.

### Statistical analysis

All statistical tests were performed using IBM SPSS Statistics 21.0 J for Windows (IBM Corp., Armonk, NY). Effects of age and gender were analyzed by two-way analysis of variance (ANOVA) with a type III sum of squares. The effects were considered significant at an alpha level of 0.05. The effect sizes (*η*^2^; not partial *η*^2^) were presented for the results of ANOVA. According to Cohen’s guideline [23], *η*^2^ values of 0.01, 0.06, and 0.14 were interpreted as small, medium, and large effects, respectively.

## Results

**Figure 2:**
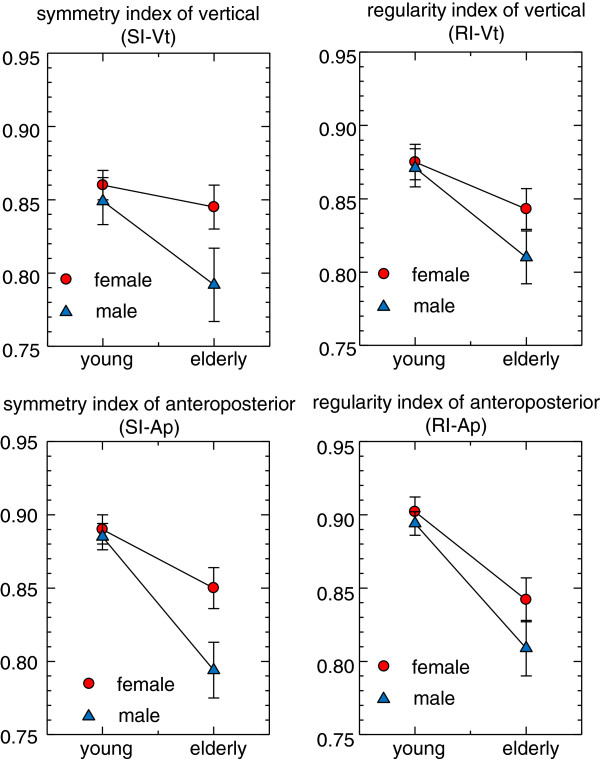
**Effects of age and gender on gait indices of vertical and anteroposterior accelerations.** Circles: mean value for the female participants; triangles: mean value for the male participants; error bars: standard error of the mean. Higher symmetry and regularity indices were observed for the younger participants when compared with the elderly participants of both genders. With regard to the gender difference, males showed less symmetry and regularity compared with females in the elderly groups.

The effects of age and gender on gait indices are shown in Figure 2 and Table 2. For both genders, young participants showed a higher symmetry and regularity than elderly participants for both vertical and anteroposterior axes. In young groups, male and female participants showed almost similar values of symmetry and regularity; however, in elderly groups, males showed lower symmetry and regularity compared with females. The results of two-way ANOVA are summarized in Table 3. For SI-Vt, both age and gender effects were significant (p = 0.022, *η*^*2*^ = 0.059 and p = 0.041, *η*^*2*^ = 0.046), although the combined effect of age and gender together was not statistically significant. The RI-Vt values were significantly affected by age (p = 0.001, *η*^*2*^ = 0.118), but the effect of gender and the interaction effect were not significant. The symmetry and regularity indices of anteroposterior acceleration (SI-Ap and RI-Ap) showed results similar to those for vertical acceleration. For SI-Ap, both age and gender differences were significant (p = 0.001, *η*^*2*^ = 0.238 and p = 0.017, *η*^*2*^ = 0.052), and the interaction effect between age and gender was statistically significant (p = 0.048, *η*^*2*^ = 0.034). The RI-Ap values were significantly affected by age (p = 0.001, *η*^*2*^ = 0.267), but the gender and the interaction effects were not significant.

**Table 2:** Symmetry and regularity indices of different age and gender groups

**Vertical acceleration**		**Symmetry index SI-Vt**		**Regularity index RI-Vt**	
**Age**	**Gender**	**Mean**	**SEM**	**Mean**	**SEM**
Elderly	Female	0.856	0.016	0.867	0.014
	Male	0.797	0.021	0.835	0.017
Young	Female	0.874	0.009	0.900	0.008
	Male	0.861	0.015	0.896	0.010
**Anteroposterior acceleration**		**Symmetry index SI-Ap**		**Regularity index RI-Ap**	
Elderly	Female	0.864	0.012	0.875	0.013
	Male	0.815	0.016	0.847	0.015
Young	Female	0.893	0.009	0.917	0.008
	Male	0.893	0.010	0.918	0.006

**Table 3:** Results of ANOVA for the effects of age and gender on symmetry and regularity of gait

	**Vertical acceleration**				**Anteroposterior acceleration**			
	**Symmetry index**		**Regularity index**		**Symmetry index**		**Regularity index**	
	**(SI-Vt)**		**(RI-Vt)**		**(SI-Ap)**		**(RI-Ap)**	
**Factor**	***p***	***η***^***2***^	***p***	***η***^***2***^	***p***	***η***^***2***^	***p***	***η***^***2***^
Age	0.022*	0.059	0.001**	0.118	0.001**	0.238	0.001**	0.267
Gender	0.041*	0.046	0.189	0.018	0.017*	0.052	0.118	0.022
Age*gender	0.179	0.020	0.287	0.012	0.048*	0.034	0.339	0.007

*p < 0.05, **p < 0.01.

*η*^2^: effect size for each factor.

## Discussion

### Gait symmetry and regularity of normal adults

Regarding gait indices of anteroposterior acceleration, elderly participants of this study showed approximate values of 0.80 - 0.86 for symmetry index and 0.84 - 0.88 for regularity index (see Table 2). Gait symmetry and regularity presented in this study were higher than those reported in the previous studies [7,19-21]. A possible reason for higher symmetry/regularity in Japanese elderly population may be a difference in physical traits. Japan is known to have the lowest obesity prevalence among developed countries [24]. Most participants of the present study were non-obese (BMI < 30 Kg/m^2^), with one exception of a young male participant (BMI = 32.6 Kg/m^2^). Some studies demonstrated that obesity affects gait pattern [25] and balance ability [26]; thus it could be assumed that obesity also affects gait symmetry and regularity.

### Effect of age on gait symmetry and regularity

In the present study, the symmetry index was affected by both age and gender, whereas the regularity index was affected only by age (see Figure 2 and Table 3). The effect sizes of age effect (approximately 0.06-0.27) were larger than those of gender effect (approximately 0.02-0.05). Previous research has reported a lack of significant effects of age on gait symmetry and regularity [27-30], opposite to the clear findings of this study (Table 3). Differences in age distribution of the sample population may possibly explain this discrepancy. Contrary to previous studies who investigated sample population continuous variation of age [27-30], the present study compared two groups of clearly different ages (20 and 70 years for the young and elderly group, respectively). Himann et al. [31] demonstrated that human gait function remains largely unchanged up to the age of 60 years, after which it is rapidly altered, explaining why studies considering a continuous age variation would fail to find a significant effect of age on gait. In fact, Kobsar et al. [18] after comparing gait symmetry of two distinguished age groups (24 and 72 years in average), found a significantly lower symmetry in elderly participants similarly to our results.

Kobsar et al. [18] also reported that a significant age effect was found only in the gait indices of anteroposterior acceleration but not in vertical and mediolateral accelerations. In this study, significant age effects were found in both vertical and anteroposterior axes. However, the effect sizes of age on the gait indices of anteroposterior acceleration were larger than those of vertical acceleration. In this respect, the present results agreed with those of Kobsar et al. [18]. However intraindividual reproducibility of gait indices of anteroposterior axis were inferior compared with those of vertical axis. Further studies are needed to determine which axis of trunk accelerometry is useful for evaluating gait symmetry and regularity.

### Effect of gender on gait symmetry and regularity

In addition to age, gender also showed a significant effect on human gait. Females usually walk slower with shorter stride length than males. Ko et al. [32] showed a significant effect of gender on kinematic features of gait, including range of motion (ROM) of hip and ankle. However, gender effect on symmetry and regularity of gait has not been sufficiently clarified yet. Previous studies [27-30] have reported an insignificant effect of gender on gait symmetry and regularity, although the detailed procedures to calculating of the gait indices differed from the present study. Our results, on the other hand, do show a significant effect of gender on gait symmetry (Table 3). However, the effect sizes of gender on gait symmetry were smaller than those of age. For the normal adult population, age is the main factor affecting gait symmetry rather than gender.

### Limitations

The short walking distance represents a limitation of this study. The participants walked 7 m and the autocorrelation function was calculated using the acceleration from the 4-s mark in the middle of each 7-m walk. The possibility that the analyzed data partially include unstable gait at the beginning and the end of the walk cannot be discarded. However, we believe that if the data would include an unstable portion of walking, the symmetry and regularity of the gait will indicate lower value. As mentioned before, the symmetry and regularity presented in this study were higher than those reported in previous studies. Furthermore, the reproducibility of the gait indices was not inferior to previously published results. This indicates that the possible effect of the unstable portion of walking on the presented results can be regarded as insignificant.

## Conclusion

The present study investigated the effects of age and gender on symmetry and regularity of gait using autocorrelation function of trunk acceleration. Significant effects of age and gender on gait symmetry were demonstrated whereas the regularity index was affected only by age. The results suggest symmetry is more sensitive to gender difference than regularity. The results also suggest that age is the main factor affecting the gait indices rather than gender if variation in age is large enough. An interaction between age and gender was significant for SI-AP; gender differences tended to be larger in elderly participants than in young participants. Although the interaction was not significant for SI-Vt, a potential combined effect of age and gender on the symmetry of gait can be presumed.

## Abbreviations

ANOVA: Analysis of variance; Ad_1_: First peak of the autocorrelation; Ad_2_: Second peak of the autocorrelation; BMI: Body mass index; ICC: Intraclass correlation coefficient; RI-Ap: The regularity index for anteroposterior acceleration; RI-Vt: The regularity index for vertical acceleration; SI-Ap: The symmetry index for anteroposterior acceleration; SI-Vt: The symmetry index for vertical acceleration.

## Competing interests

The authors declare that they have no competing interests.

## Authors’ contributions

HK performed statistical analysis, and drafted the manuscript and study design. WK performed measurements. TK participated in study design and obtained final approval. All authors read and approved the final manuscript.
